# [Corrigendum] p53 positively regulates the expression of cancer stem cell marker CD133 in HCT116 colon cancer cells

**DOI:** 10.3892/ol.2026.15452

**Published:** 2026-01-08

**Authors:** Xia Chen, Hua Guan, Xiao-Dan Liu, Da-Fei Xie, Yu Wang, Teng Ma, Bo Huang, Ping-Kun Zhou

Oncol Lett 16: 431-438, 2018; DOI: 10.3892/ol.2018.8619

Following the publication of the above article, an interested reader drew to the authors' attention that the control GAPDH western blots featured in Fig. 2A on p. 434 and Fig. 3A on p. 435 were strikingly similar, albeit they appeared at different sizes and the experimental contexts of these two figures were different.

After having re-examined the original data for these figures, the authors have realized that the GAPDH western bands for Fig. 2A were inadvertently chosen incorrectly. The revised version of Fig. 2, now showing the correct data for the control western blots in Fig. 2A, is shown below. Note that this error did not have a significant impact either on the results or the conclusions reported in this paper. All the authors agree to the publication of this corrigendum. Furthermore, the authors are grateful to the Editor of *Oncology Letters* for allowing them the opportunity to publish this corrigendum, and they apologize to the readership for any inconvenience caused.

## Figures and Tables

**Figure 2.**
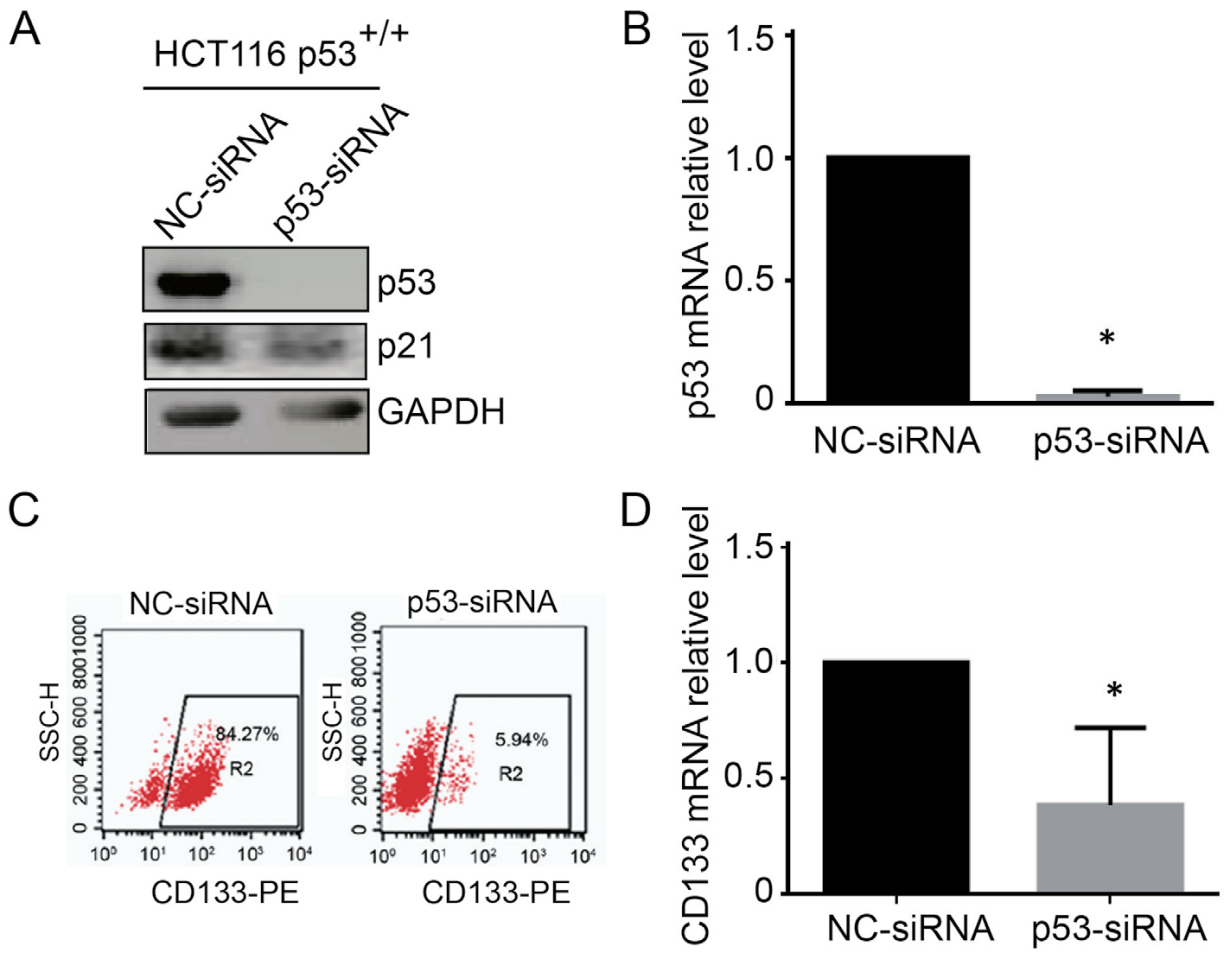
Effect of p53 silencing on CD133 expression in p53^+/+^ HCT116 cells. p53^+/+^ HCT116 cells were transfected with either a p53-specific siRNA or a negative control siRNA (NC). (A) Western blot analysis for p53 and p21 protein expression levels. (B) RT-qPCR analysis for p53 mRNA expression. *P<0.01. (C) Representative results of flow cytometric analysis for expression of the cancer stem cell marker CD133. siRNA-mediated p53 silencing resulted in a decreased % of CD133-positive cells in the p53^+/+^ HCT116 cell line. (D) RT-qPCR analysis for CD133 mRNA expression. siRNA-mediated p53 silencing resulted in decreased mRNA levels of CD133 in the p53^+/+^ HCT116 cell line. *P<0.01. si, small interfering; NC, negative control; RT-qPCR, reverse transcription-quantitative polymerase chain reaction.

